# “CAREGIVING MADE ME GRATEFUL”: THE UNEXPECTED BLESSINGS OF DEMENTIA CAREGIVING

**DOI:** 10.1093/geroni/igad104.0142

**Published:** 2023-12-21

**Authors:** Jordan Lewis

**Affiliations:** University of Minnesota Medical School, Duluth campus, Duluth, Minnesota, United States

## Abstract

There is a cultural value among Alaska Natives to care for their Elders and this commitment applies to all Elders, regardless of health status or cognitive health. Caregiver duties can lead to stress, isolation, and depression that negatively impact their overall health and wellbeing. Interviews were conducted with 22 Alaska Native caregivers for Alaska Natives with Alzheimer’s Disease and Related Disorders in rural and urban communities across Alaska. Interviews lasted approximately 60 min and were transcribed verbatim. We examined the caregivers’ experiences of ADRD and the unexpected blessings associated with being a caregiver. Alaska Native caregivers shared the challenges associated with caregiving duties, and the lack of education, awareness, and available resources exacerbated their stress and challenges. As their family member experienced changes related to the ADRD, the blessings took different forms and served different purposes. Spending more time with their family member, remembering family history, engaging in generative behaviors, and strengthening intergenerational relationships were the most frequently discussed blessings. Focusing on these blessings enabled caregivers to continue to find meaning in this role and carry on their cultural values and traditions. This study shifts the focus of caregiving research to highlight the unexpected blessings. The focus on strengths of caregiving and sharing these stories has the potential to provide current and future caregivers to step into this role with a different perspective, or at least not just hear of the challenges and barriers associated with ADRD caregiving.

